# Elasto-plastic analysis and optimal design of composite integral abutment bridge extended with limited residual plastic deformation

**DOI:** 10.1038/s41598-023-32787-y

**Published:** 2023-04-04

**Authors:** Majid M. Rad, Ferenc Papp, Sarah K. Ibrahim, János Szép, Dániel Gosztola, Dániel Harrach

**Affiliations:** Department of Structural and Geotechnical Engineering, Széchenyi István University, Győr, 9026 Hungary

**Keywords:** Engineering, Civil engineering

## Abstract

Due to the growing significance of structural theories concerning the composite structure analysed and designed plastically, this paper introduces a new optimisation method for controlling the plastic behaviour of a full-scale composite integral abutment bridge by employing complementary strain energy of residual forces that existed within the reinforcing rebars. Composite bridges are structures made of components such as steel and concrete, which are frequent and cost-effective building methods. Thus, various objective functions were used in this work when applying optimum elasto-plastic analysing and designing the composite integrated bridge structure that was tested experimentally in the laboratory. In contrast, the plastic deformations were constrained by restricting the complementary strain energy of the residual internal forces aiming to find the maximum applied load and the minimum number of steel bars used to reinforce the concrete column part of the structure. The numerical model employed in this paper was validated and calibrated using experimental results, which were considered inside ABAQUS to produce the validated numerical model, using concrete damage plasticity (CDP) constitutive model and concrete data from laboratory testing to solve the nonlinear programming code provided by the authors. This paper presents a novel optimization method using complementary strain energy to control the plastic behaviour of a full-scale composite integral abutment bridge, with the original contribution being the incorporation of residual forces within reinforcing rebars to limit plastic deformations. Following that, a parametric investigation of the various optimisation problems revealed how models performed variously under different complementary strain energy values, which influenced the general behaviour of the structure as it transitioned from elastic to elasto-plastic to plastic; also results showed how the complementary strain energy value is connected with the amount of damaged accrued in both concrete and steel.

## Introduction

Because of their remarkable aesthetic appearance and effective structural competency, steel–concrete composite bridges are widely employed across the globe. Composite bridges made of RC slabs on top of the steel beams can be efficiently designed to maximise the advantages of both the steel beams and the concrete slabs, including increased strength, ductility, stiffness, resistance to seismic loadings, material utilisation, and fire resistance, as compared to standard RC bridges and normal steel bridges. As conducting full-scale tests on steel–concrete composite bridges is expensive and time-consuming, there is a lack of test data for various bridge designs. Constant changes in bridge cross-sections, component material strengths, and applied loads are partially to blame for the lack of test data. Codes of practice for steel–concrete composite bridges today rely heavily on results from miniature and full-scale examinations of bridges and bridge components^1^. To fully comprehend the security and functionality, Lu et al.^2^ offered model testing and numerical finite element analysis (FEA) on the mechanical behaviour of a typical composite bridge at a scale of 1:30. Additionally, Wegmuller^3^ investigated the post-elastic behaviour and the effect of certain design parameters on bridge response when studying the overload behaviour of composite steel–concrete highway girder bridges, detailing a nonlinear finite element analysis procedure that allows the determination of the state of stress and deformation under given levels of overload. At the same time, Wegmuller and Amer^4^ studied the post-elastic response and overload behaviour of a composite beam-slab type highway bridge using nonlinear finite element analysis. Composite space trusses are just one type of composite construction that could benefit from the nonlinear finite element (FE) programme suggested by Sebastian and McConnel^5^.

Slab-on-I-steel girder composite bridges are still a popular design choice. To meet the design requirements for the ultimate limit states of these sorts of bridges, Kennedy et al.^6^ estimated the most likely yield-line patterns of failure for much wider composite bridges based on research work and laboratory test findings on composite bridge models. Feng et al.^7^ also demonstrated how fibre-based analysis can be used to foretell the nonlinear response of columns in a reinforced concrete bridge. In addition, various circular and square columns were analysed by Mondal and Prakash^8^, who developed a nonlinear FE model to foretell the behaviour of RC bridge columns subjected to coupled torsion and axial compression. Lastly, McCarthy et al.^9^ created computer software to elasto-plastically analyse composite steel–concrete buildings utilising the finite element approach. Tan et al. measured the damage in the primary load-bearing components of a composite slab-on-girder bridge construction, comprised of a reinforced concrete slab and three steel I beams^10^.

To learn more about the inelastic behaviour of concrete, structural steel, and longitudinal and transverse steel bars, Zhang et al.^11^ used finite element analysis to conduct parametric research on a composite column. Cross-sections of composite columns in bridge piers and buildings, structural details of standard steel and composite bridge pier columns, ultimate strength, stiffness, and ductility of composite piers are all taken into account, as detailed by Kitada^12^.

In general, plastic analysis and design of structures built of elastoplastic materials have risen to prominence in structural theory, and the methods and results of their solution have been widely implemented in reality. Using the plastic reserve of a structure, the plastic limit theorems lay the groundwork for the effective implementation of numerous optimization strategies, leading to substantial savings. There is a trade-off for this advantage, however, as plastic deformations, which can be accumulated and persist even after unloading, typically occur in the structure as a result of employing plastic procedures. Elastoplastic analysis and design, therefore, typically necessitates consideration of inelastic deformations that may arise from single- or multi-parameter loadings^13^.

A shakedown analysis and optimal shakedown design of elasto-plastic trusses were proposed in 1997 by Kaliszky and Logo^14^ for use under multi-parameter static load. The plastic behaviour of the truss is constrained by placing bounds on the complementary strain energy of the residual forces and the residual deformations. The complementary strain energy of the remaining internal forces is restricted to limit permanent deflections, and the volume of the structure is taken into account as an objective function. Then, in 1999^15^, Kaliszky and Logo introduced a designing process for an optimised strengthened truss, intending to strengthen the truss with additional elasto-plastic supports and/or bars, guaranteeing that the truss can support the loads without undergoing additional plastic deformations, all while keeping the cost of the strengthened truss to a minimum. The bar-optimised cross-sectional area of a steel structure subject to strength, stiffness, and stability restrictions was also supplied by Gervyte and Jarmolajeva^16^, who developed a mathematical model for this problem. In addition, Movahedi Rad et al.^17^ recently presented a novel optimisation strategy for regulating the plastic behaviour of haunched reinforced concrete (RC) beams through the utilisation of complementary strain energy of residual forces within the steel rebars to establish the maximum loading or steel minimum volume required for reinforcing beams.

Composite integral abutment bridges are critical structures used in civil engineering, and the plastic behaviour of these structures is essential for their overall performance and durability. The significance of this study is to introduce a new optimisation method that controls the plastic behaviour of composite integral abutment bridges by utilizing the complementary strain energy of residual forces in the reinforcing rebars. The novelty of this study is in the application of an optimal elasto-plastic analysis and design approach to composite integral abutment bridges and the incorporation of the complementary strain energy of residual internal forces in the design process. where the main objective of this study is to optimize the elasto-plastic behaviour of a full-scale composite integral abutment bridge by controlling the plastic deformation of the structure. This was considered by applying an optimal elasto-plastic analysis and design approach and restricting the plastic deformations by controlling the complementary strain energy of the residual internal forces. Aiming to find the maximum applied load and the minimum number of steel bars required to reinforce the concrete column part of the structure while investigating the effect of different complementary strain energy values on the overall behaviour of the structure as it transitions from elastic to elasto-plastic to plastic.

As this study aims to limit the plastic behaviour of a steel–concrete composite integral abutment bridge by proposing a unique optimum elasto-plastic analysis and design technique that focuses on controlling steel bars’ residual plastic deformation used to reinforce the bridge structure and employing the complementary strain energy of residual force that considers a world-wide displacement restriction. Because of the model’s complexity, it was primarily necessary to validate the numerical models based on laboratory test results which were obtained by testing a full-scale composite integral abutment bridge model. Only then the second step could be followed which includes the analysis of different components of the models and the study of the different constraints. The concrete damage plasticity CDP model was utilized to calibrate the numerical model representing the created damage in the concrete^18^. Moreover, the used method is extended from the truss study made originally by Kaliszky and Logo^19^ and then extended to include reinforced concrete haunched beams by Movahedi Rad et al.^17^ as it is assumed by the authors that this is a novel study concerning this complicate model and optimization problem that took into consideration finding the optimal applied load and the optimal steel bars number. In order to optimise the imposed plastic load and the number of steel bars reinforcing the column part of the abutment bridge construction, different objective functions were explored. Complementary strain energy was presumed as a constraint to regulate the residual plastic behaviour of these steel rebars. The Abaqus Scripting Interface (ASI) is a Python-based interface that allows users to automate ABAQUS simulations by writing scripts. In this study, ASI was used to integrate the optimization algorithm into the ABAQUS model. Specifically, a programming script that defined the nonlinear optimization problem, constraints, and objective function, was considered. Residual stresses were calculated after running the code for all the increments in order to determine the optimum load value, and the corresponding strain energy was calculated and matched to the permissible value stated in the code. Another code, on the other hand, was created to compute the optimal amount of reinforcing steel bars necessary for individually specific complementary strain energy, taking into account a design problem. The impact of complementary strain energy on the limited plastic load capacity and the quantity of reinforcing steel is then investigated.

After this introduction, the limited plastic deformation clarification was presented in Section “Application of the theory of limited residual plastic deformation to steel bars”. The experimental work details are presented in Section “Experimental work”, while in Section “Finite element model validation” the numerical modelling details are explained. In addition, Sections “Analysis optimization problem” and “The problem of the design optimization” reveal the optimization problems formulations and a results summary and clarifications. Lastly, Section “Conclusions” illustrates the conclusions.

## Application of the theory of limited residual plastic deformation to steel bars

This theory takes into consideration applying a limitation on the plastic deformation created within steel bars by defining the complementary strain energy which was applied and developed by different previous studies ^17,20–22^. Using the Euler notation, consider a body constructed of an elastic–plastic material that is not affected by time or temperature and has a volume ${V}_{0}$ and a surface $S$. While a portion of $S,$ ${S}_{q}$ is subjected to quasi-static surface tractions, the remaining portion ${S}_{u}$ is subject to zero surface displacements. The following values are described^23^ at time $t$ for surface tractions ${q}_{i }(t)$:${\sigma }_{ij}\left(t\right)$= actual stresses${\epsilon }_{ij}\left(t\right)$ and ${u}_{i}\left(t\right)$= actual strains and displacements${\sigma }_{ij}^{el}\left(t\right)$ = fictitious stresses that would occur if the material were purely elastic${\epsilon }_{ij}^{el}\left(t\right)$ and ${u}_{i}^{el}\left(t\right)$= fictitious elastic strains and displacements corresponding to ${\sigma }_{ij}^{el}\left(t\right)$

In addition, the following self-stress distributions are discussed:${\sigma }_{ij}^{R}(t)$ = actual residual stress distribution${\tilde{\sigma }}_{ij}^{R}$ = any arbitrary, time-independent self-stress distribution

As indicated in Eq. (1), the actual strain is split into elastic and plastic components. The constitutive law links the elastic strain portions to actual stresses, according to the latest notation,

1 $${\epsilon }_{ij}={\epsilon }_{ij}^{el}+{\epsilon }_{ij}^{pl}$$

2 $${\epsilon }_{ij}^{el}={C}_{ijkl}{\sigma }_{kl}^{el}.$$

The elastic tensor ${C}_{ijkl}$ is determined by the related flow rule, and the plastic strain ${\epsilon }_{ij}^{pl}$ is specified by the accompanying flow rule,

3 $${\epsilon }_{ij}^{pl}=\lambda \frac{\partial f}{\partial {\sigma }_{ij}}, \dot{\lambda }\ge 0 \mathrm{if} f=0 \mathrm{and} \dot{f}=0, \mathrm{otherwise} \dot{\lambda }=0,$$

Here ${f(\sigma }_{ij})$ is the yield function and ${f(\sigma }_{ij})$= 0 defines a convex surface in the stress space.

Finally, the actual stresses ${\sigma }_{ij}\left(t\right)$*,* the fictional elastic stresses ${\sigma }_{ij}^{el}\left(t\right)$*,* and the actual residual stresses ${\sigma }_{ij}^{R}$ must satisfy the following relation:

4 $${\sigma }_{ij}\left(t\right)= {\sigma }_{ij}^{el}\left(t\right)+ {\sigma }_{ij}^{R},$$

Here ${\sigma }_{ij}^{el}\left(t\right)$ is related to the fictitious elastic strain ${\epsilon }_{ij}^{el}(t)$ by the constitutive law,

5 $${\epsilon }_{ij}^{el}\left(t\right)={C}_{ijkl}{\sigma }_{kl}^{el}\left(t\right).$$

Estimate the overall complementary plastic work ${W}_{p}(\tau )$ produced along a load route from $t$ = 0 to $t$ = $\tau$. This research might be utilised to assess an elasto-plastic body's plastic performance and overall plastic deformation. Capurso^24^ and Capurso et al.^25^ suggested the next theorem to find its upper bound.

Whether any time-independent self-stress distribution ${\tilde{\sigma }}_{ij}^{R}$ can be discovered that meets the condition:

6 $$f\left({\sigma }_{ij}^{E}\left(t\right)+ {\tilde{\sigma }}_{ij}^{R}\right)\le 0,$$

is met in *V* at any time $t\le \tau$, then the following condition sets the upper constraint on the total complementary plastic work:

7 $${W}_{p}\left(\tau \right)\le \frac{1}{2}\int {C}_{ijkl} {\tilde{\sigma }}_{ij}^{R} {\tilde{\sigma }}_{kl}^{R} dV$$

It should be highlighted that the bound can be enhanced^5^ by selecting the right value for ${\tilde{\sigma }}_{ij}^{R}$. Take a look at Eq. (7) to see how the bound on complementary plastic work is defined. Impose a properly determined allowed restriction ${W}_{p0}$ on the plastic work ${W}_{p}$ to prevent excessive plastic deformations. The real residual stresses define the bounds of the plastic deformations; in other words, it is assumed that:

8 $${\tilde{\sigma }}_{ij}^{R}\equiv {\sigma }_{ij}^{R}.$$

This presumption gives a reasonable upper bound and helps us to formulate the problem properly. In such case, the plastic deformation constraint will like the following:

9 $${W}_{p}(\tau )=\frac{1}{2}{\int }_{{V}_{0}}^{V}{C}_{ijkl} {\sigma }_{ij}^{R} {\sigma }_{kl}^{R}dV- {W}_{p0} \le 0$$

As a result, using constraints for such energy quantities, a computational method was devised to describe the complementary strain energy of residual forces as a general evaluation of the plastic behaviour of structures where residual deformations must be controlled^14,15,26^. For the example of bar elements, Eq. (9) was created, and the residual forces are used to determine the complementary strain energy as follows:

10 $${W}_{p}=\frac{1}{2\mathrm{E}} {\sum }_{\mathrm{i}=1}^{\mathrm{n}}\frac{{l}_{i}}{{A}_{i}}{ N}_{i}^{{R}^{2}}\le {W}_{p0},$$

where ${W}_{p0}$ is an appropriate allowable energy value for ${W}_{p}$. Also, ${l}_{i}, (i=1, 2, \dots , n)$ denotes the length of the members creating the bar elements, while the cross-sectional area of the bar elements is characterised by , ${A}_{i}, (i=1, 2, \dots , n)$ while ${N}_{i}^{R}$ denotes the residual force of the bar members and $\mathrm{E}$ is Young's modulus of the material of bars. By using Eq. (10), the plastic deformations of bar elements are contained as an appropriate limit value ${W}_{p0}$.

Furthermore, when the load ${P}_{0}$ is applied, the internal plastic force ${N}^{pl}$ and the internal elastic force ${-N}^{el}$ indicate the residual forces ${N}^{R}$ that remain in the structure after the unloading is complete:

11 $${N}^{R}={N}^{pl}-{N}^{el}$$

where:

12 $${N}^{el}={F}^{-1}{G}^{T}{K}^{-1}{P}_{0}$$

The flexibility matrix is designated as *F*, whereas the geometry matrix is designated as *G*, and the stiffness matrix is designated as *K*. To limit the plastic deformation created within steel bars, the boundary of plastic deformation utilising complementary strain energy is considered to steel bars situated inside the reinforced concrete column part of the bridge structure. Internal forces developed in concrete, on the other hand, are not included in the optimization process due to its low influence in tension in comparison to steel, while it is recognised that steel endures more tension strain than concrete, which can cause an early collapse of concrete subjected to tension.

The main objective of the optimization method used in this research is to understand and control the plastic behaviour of the structures as it is the stage that should be avoided in the real age of the structure. When understanding this behaviour, the process of controlling it would be relatively easy, and in our research, the complementary strain energy introduced above was used to control the plastic behaviour of the structure. This theory is applied to steel elements that are yielded, and since steel is represented in the composition of our structure in two forms which are steel beam and reinforcing steel bars, however, the steel beam was excluded because it did not yield and the theory was considered to the steel bars used as reinforcement in the column section of the bridge structure as they were the only steel elements that yielded. Thus, taking into account these yielded steel elements, the optimal solution was applied as an analytical problem and another as a design one.

## Experimental work

**Figure 1:**
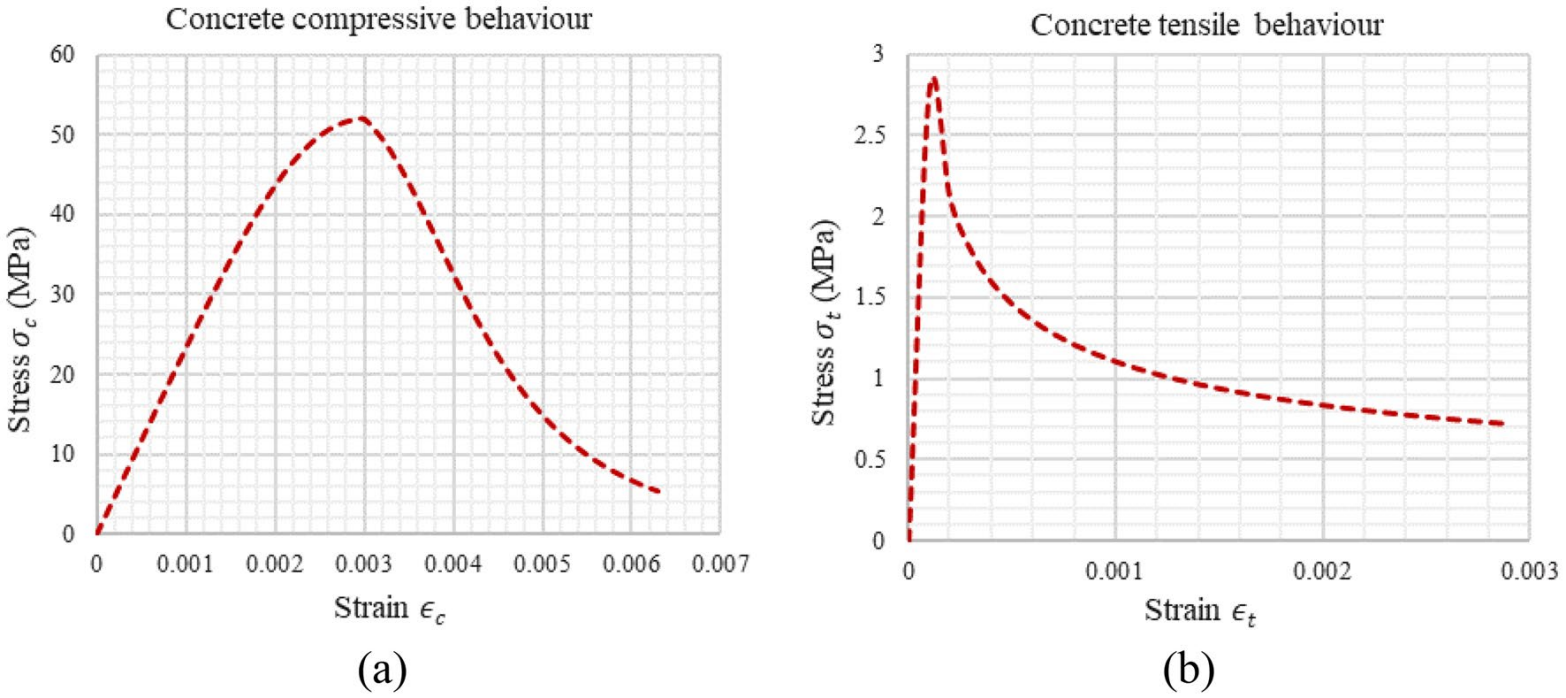
Experimental properties of concrete in: (**a**) Compression, (**b**) Tension.

Concrete bridges are susceptible to plastic behaviour under high loads and severe loading conditions, which can lead to significant damage or collapse. Usually, to contain such failures, retrofitting and strengthening techniques can be employed to enhance the ductility and energy dissipation capacity of the bridge^27^. However, avoiding reaching the plastic behaviour can be obtained by optimal plasticity control. In this research, the composite integral abutment bridge model was prepared and tested experimentally in Széchenyi István University laboratory according to the dimensions and details presented in this section where the model consists of two materials, steel and concrete. Steel is represented by reinforcement bars, which have experimentally obtained properties illustrated in Table 1, and steel beam HEA 450 that supports the concrete deck. Moreover, concrete properties were measured experimentally in the laboratory. Additionally, four samples for each test were created and evaluated after 28 days of curing, resulting in average concrete characteristics in compression and tension. These measurements were done experimentally in the lab for both concrete tensile behaviour and compressive behaviour as shown in Fig. 1.

**Table 1:** Properties of steel reinforcement.

Specifications	Yield strength (MPa)	Ultimate tensile strength (MPa)	Elastic modulus (MPa)
$\upphi$ = 16 mm	435	494	210,000
$\upphi$ = 10 mm	478	544	210,000
$\upphi$ = 6 mm	480	546	210,000

**Figure 2:**
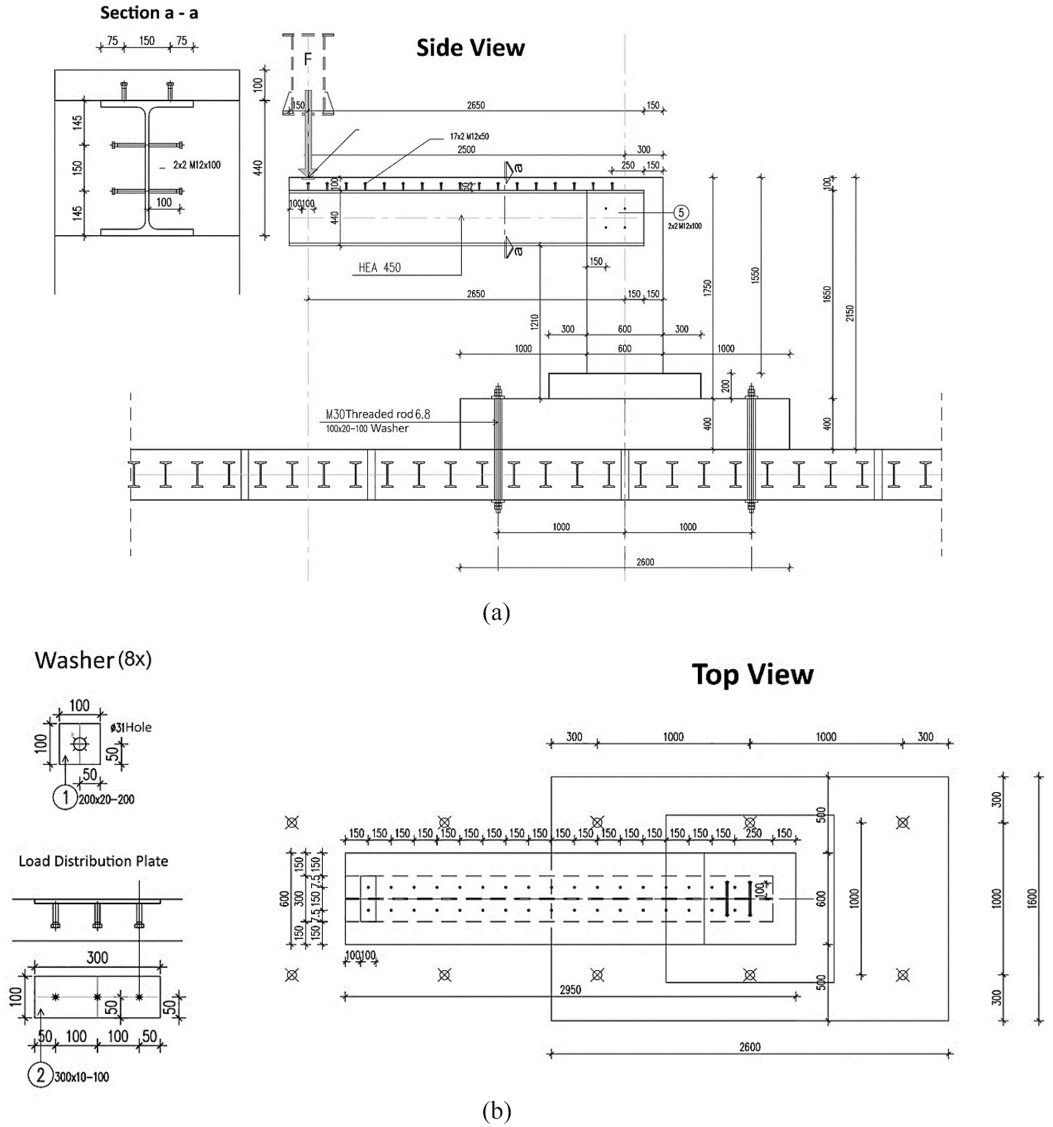
Geometry of the specimen: (**a**) Side view, (**b**) Top view.

**Figure 3:**
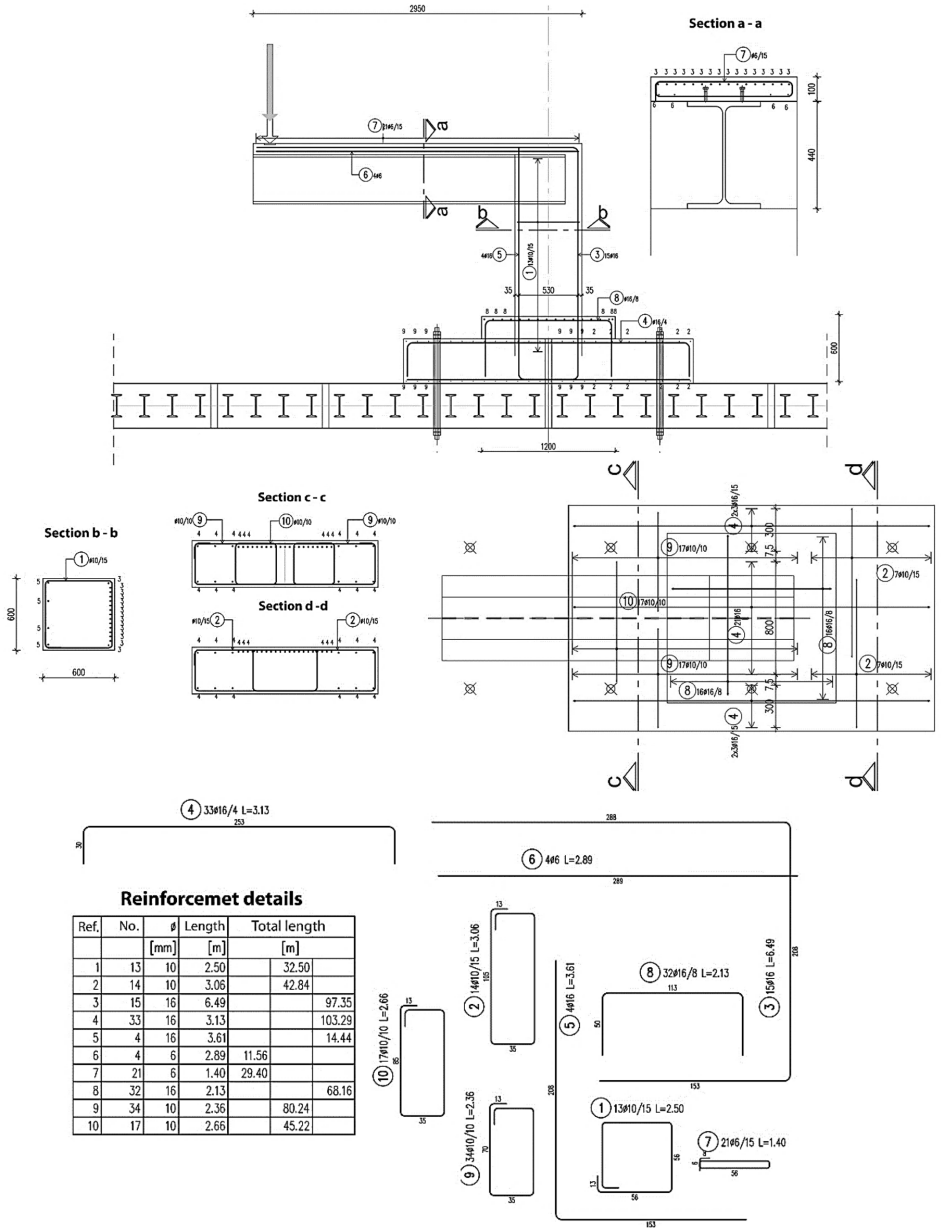
Reinforcement details.

**Figure 4:**
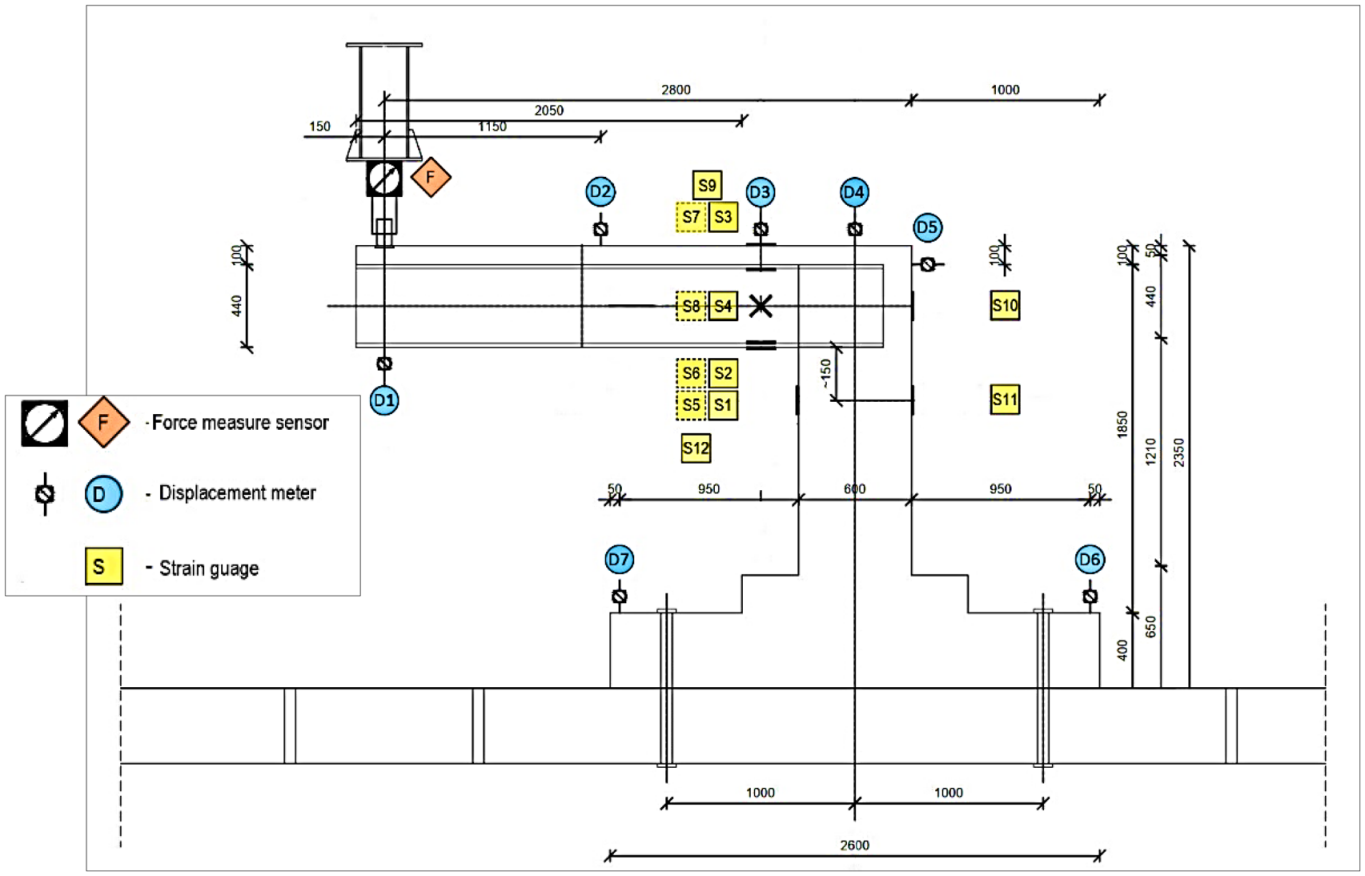
Sensors types and locations.

**Figure 5:**
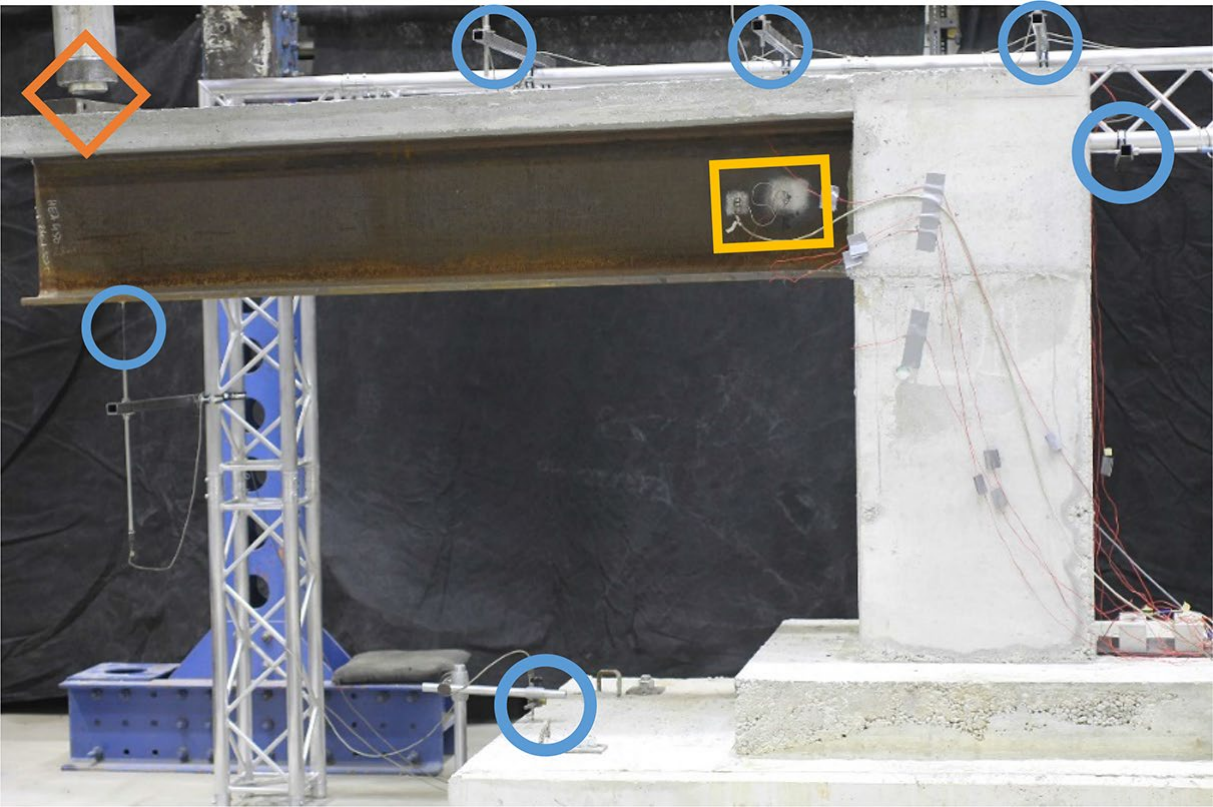
Sensors Installation during the experiments work.

Figures 2 and 3 display the dimensions and reinforcement details of the specimen used in the experimental test, including information on the length, width, and height of the specimen, as well as the thickness of the concrete. Furthermore, Fig. 3 provides details on the type and amount of reinforcing steel used, including the bar diameter, spacing, and configuration. Moreover, Figs. 4 and 5 show the types and locations of the sensors used in the experimental test for load, displacement and strain. These figures include information on the number of sensors used and their location in the specimen, as well as details on the type of sensors used.

**Figure 6:**
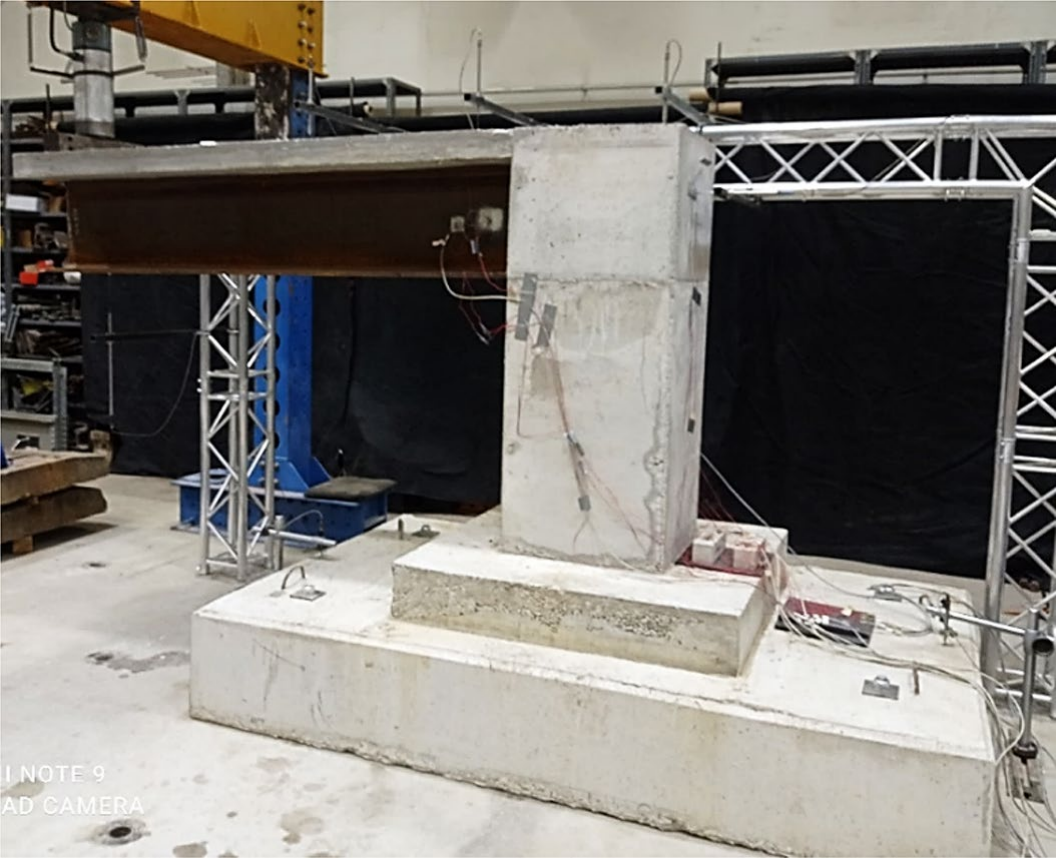
Composite integral abutment bridge structure set up.

The structure was essentially attached to the laboratory ground slab with four M30 threaded rods and washers, whereas the steel beam was attached to the concrete components of the structure with bolts, as indicated in Fig. 2. After 28 days of curing, specimen testing was carried out in the laboratory to determine the structure’s behaviour with regard to load–deflection, failure mode, and cracking pattern relation. The test was carried out by applying one concentrated monotonic loading at the end of the cantilever part up to failure, with a rigid plate used to distribute the applied load as shown in Fig. 6.

## Finite element model validation

**Figure 7:**
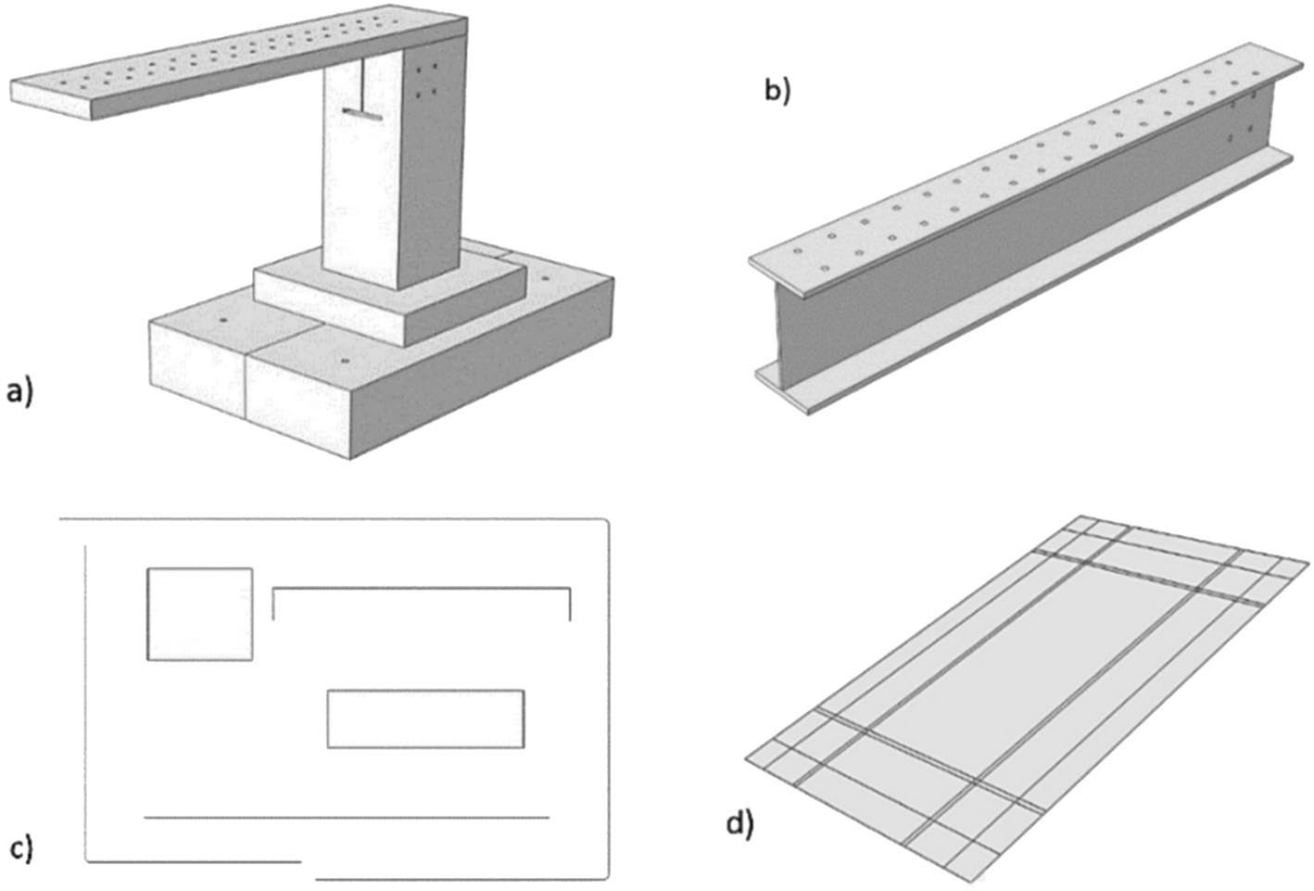
Numerical model components: (**a**) concrete part, (**b**) Steel beam, (**c**) Reinforcement bars, (**d**) Rigid plate.

The Three-Dimensional finite element model was validated by ABAQUS employing the experimental test results. Generally, the bridge model consists of four different parts as shown in Fig. 7 where the finite element model was created by steel beam, steel reinforcement materials and concrete. Basically, a 3D four nodes solid element (C3D4: four-node linear tetrahedron element) was used to represent both concrete and steel beam, to simulate the connection between the longitudinal and transverse reinforcements and concrete, an embedded area was used while representing the reinforcement bars as a 3D beam element with a 2-node linear beam in space (B31: Timoshenko beam). Moreover, the loading plate was modelled using shell elements (S4R: four-node doubly curved thin or thick plate) while the plate holding the structure from below was represented by a 3D rigid shell model using R3D4 elements (four-node 3D bilinear rigid quadrilateral elements) depending on the experimental test results where the displacement in this plate was zero so a rigid shell was considered in order to reduce the time of calculation. Then, after sensitivity analyses were performed, the CDP plasticity model that was used to represent concrete behaviour is assumed with the parameters given in Table 2 bearing in mind that Young's modulus of concrete equals *E*_0_ = 34,000 N/mm^2^ and Poisson’s ratio equals *v* = 0.2. Calibrating the concrete damage plasticity (CDP) model based on experimental results typically involves adjusting the material parameters of the model until it closely matches the behaviour observed in the experimental data. In this study, concrete data from laboratory testing was used to calibrate the CDP model. Specifically, the predicted response of the CDP model was compared to the measured response of the concrete specimens while the material parameters of the CDP model, such as the tensile and compressive strengths of concrete were adjusted until a satisfactory match was achieved between the predicted and measured response. The mechanical properties tests of specimens were used to get the concrete damage plasticity data, which includes tensile cracking and compressive crushing that represent the material's failure mechanisms.

**Table 2:** Inserted CDP data for concrete.

Dilation angle	Eccentricity	${f}_{b0}/{f}_{c0}$	$K$
31	2	1.16	0.67

**Figure 8:**
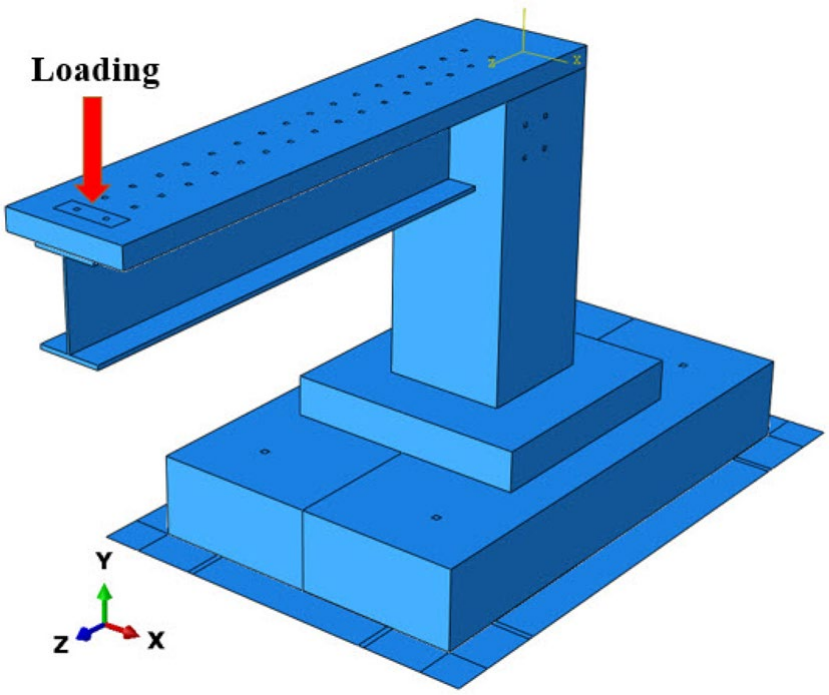
Loading condition.

The boundary conditions of the calibrated composite bridge were chosen to match laboratory test settings, the concrete and the steel beam were connected by applying beam contact -which is available inside ABAQUS- to the bolts existing between, while the structure was connected to the supporting rigid plate by applying tie contact on the threaded rods, in meantime, the surface to surface hard contact was used to connect the other parts together. To obtain the equivalent experimental circumstances, a vertical concentrated load was applied at the model's point load as clarified in Fig. 8 as this load was distributed by using the coupling effect, also, in this numerical simulation, large deformations were taken into account, as a result of which the stiffness changes during the simulation, which is automatically corrected by the software.

**Figure 9:**
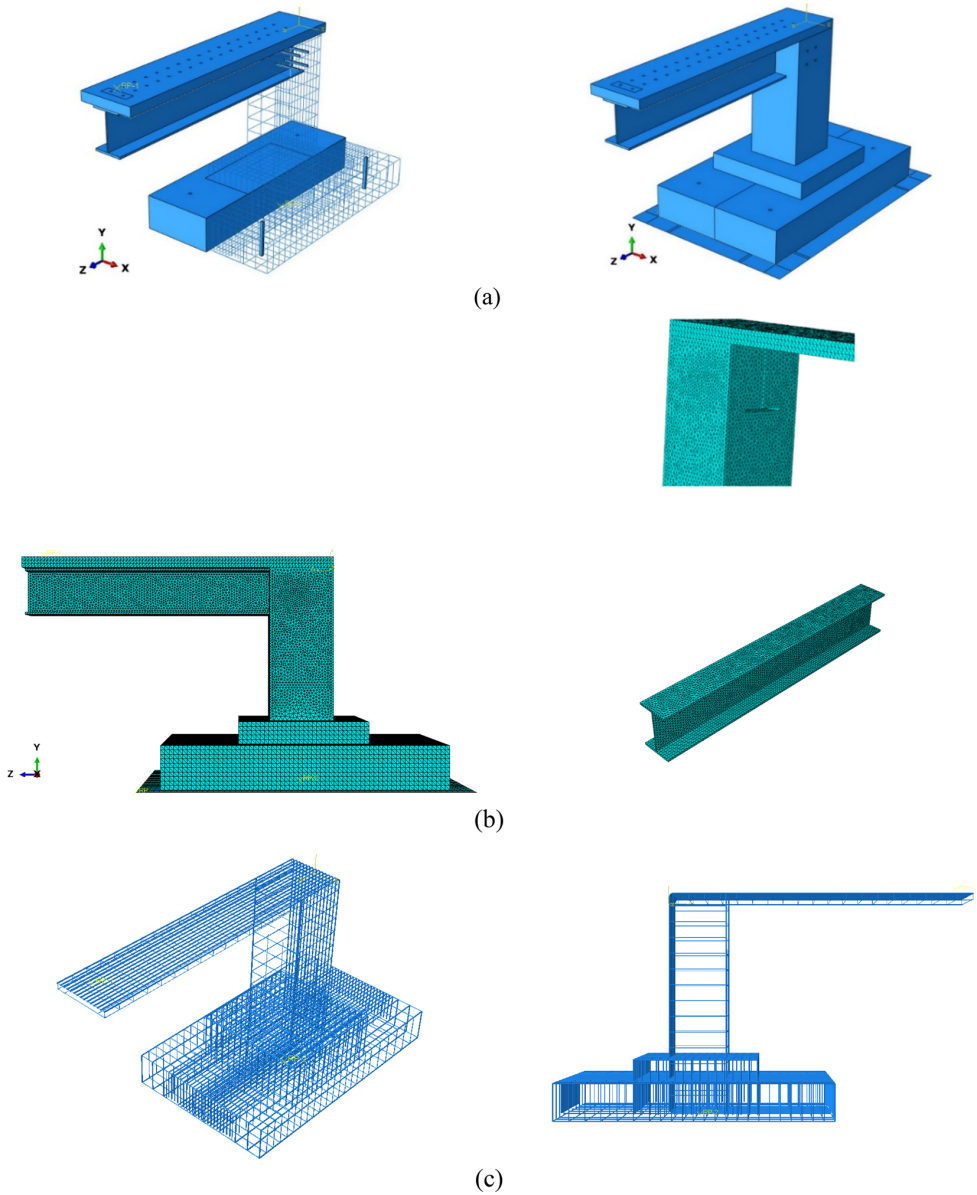
Numerical details of the model: (**a**) model components, (**b**) Meshing, (**c**) Reinforcement cage.

Besides, each part of the model is located in its own coordinate system and is independent of the other parts of the model. Although the model consists of different parts, overall, it can be considered a unit model, Fig. 9 shows numerical details of the integrated bridge model. The influence of mesh size on accuracy and computation time was examined, and the optimal mesh size was then employed to ensure correct results. The total number of elements in the structure was roughly 162,032.

**Figure 10:**
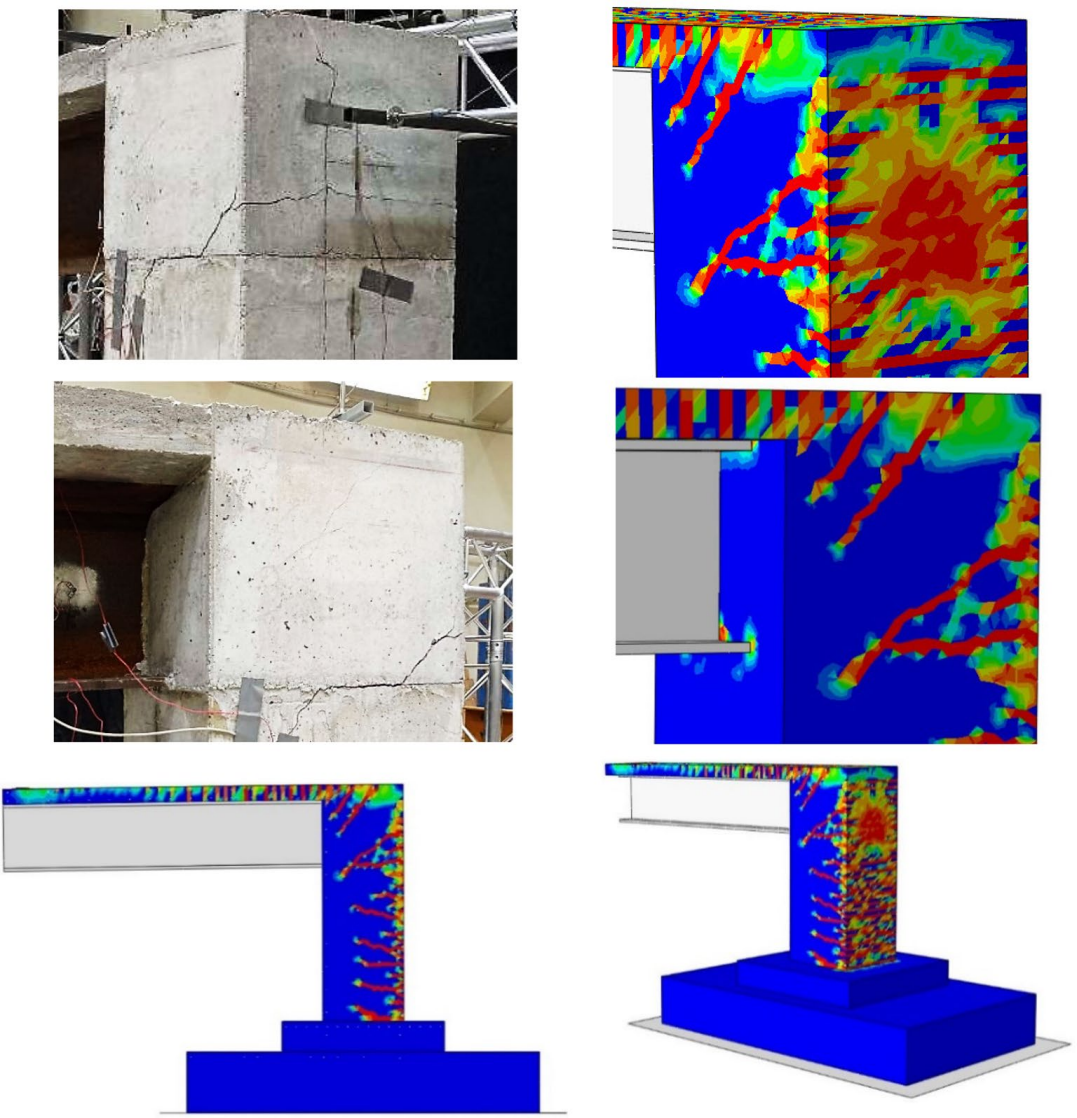
Experimental and numerical cracking patterns.

**Figure 11:**
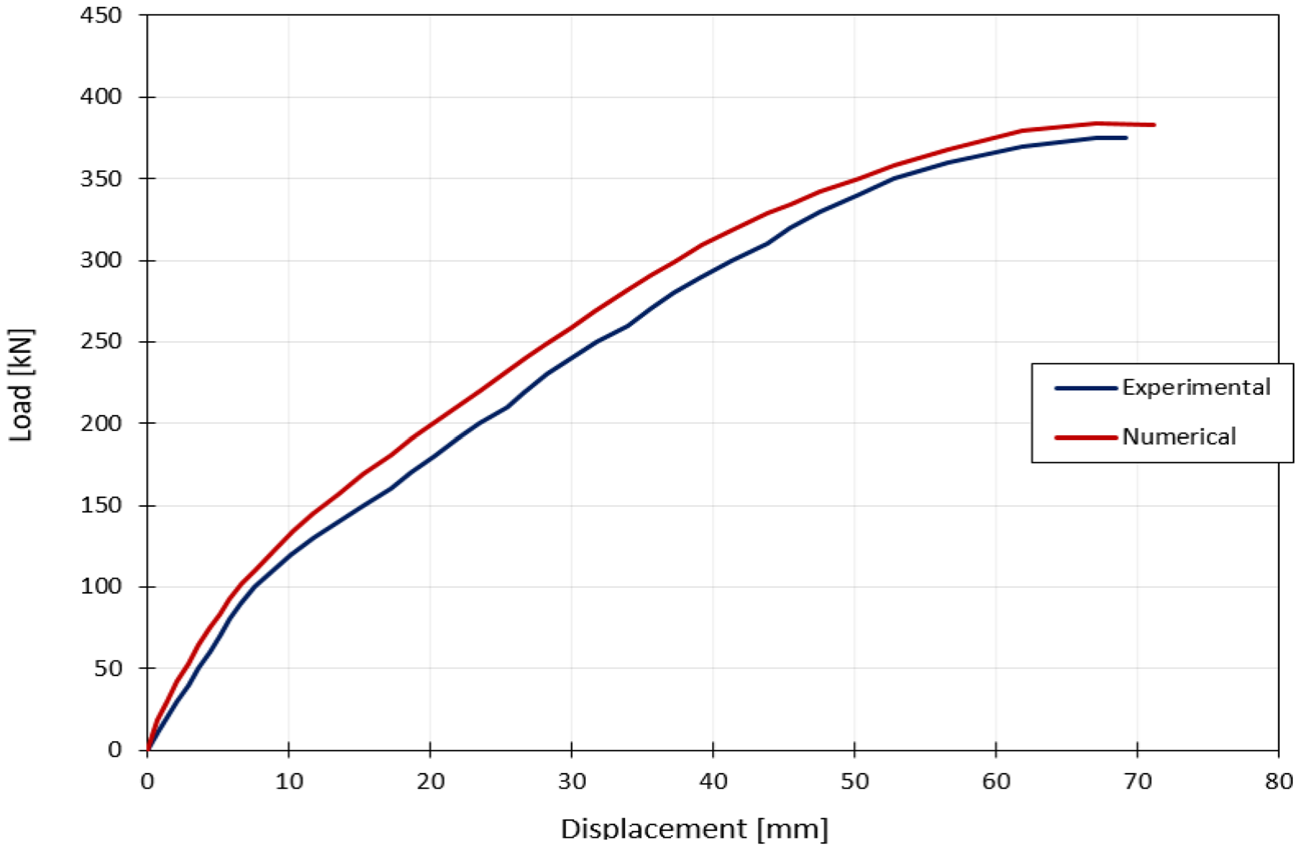
Load–displacement comparison.

So as to validate the bridge model, a numerical process was held in order to obtain the numerical results and then to be confirmed with experimental results. Basically, it was discovered by comparing the numerical and experimental data that the crack patterns of the numerical structure occur at nearly identical locations as in the experimental model that major and minor cracks are distributed at the failure area, where the pattern of concrete tension damage in these two situations are very comparable, as shown in Fig. 10, and the failure is located in the column part, as the severity of damaged regions varies by colour, with blue indicating undamaged parts and red indicating entirely damaged areas. Additionally, the load–deflection curves from the experimental and numerical tests are comparable, with the ultimate load capacity measured numerically being around 388 kN and the ultimate load capacity tested experimentally being about 380 kN, as seen in Fig. 11. Thus, it can be concluded that the numerical modelling and laboratory results are showing a great similarity.

It is noted from the presented results that the presence of the steel beam was effective in preventing failure in the deck part of the bridge despite the appearance of cracks in the concrete slab. Therefore, the stresses accumulated in the column section which is responsible for transferring the loads from the bridge deck to the base. In that part, the metal bars were under high stresses, which caused their yielding after cracking of the concrete, as the column part suffers from tensile stresses in which the concrete is weak. Despite the congestion of the reinforcing bars in the failed part where 15 bars with a diameter of 16 mm were used, the high stresses caused the yielding of these bars and consequently the failure of the structure at a load equal to 380 kN approximately.

Moreover, after analysing and comparing the sensors' results, the strain and displacement values were examined in different parts of the elements to assess the distribution of the load. The results of this analysis showed that the stress and displacement values were very similar if compared with the experimental results in different parts of the specimen, indicating that the calibrated model was obtained.

**Figure 12:**
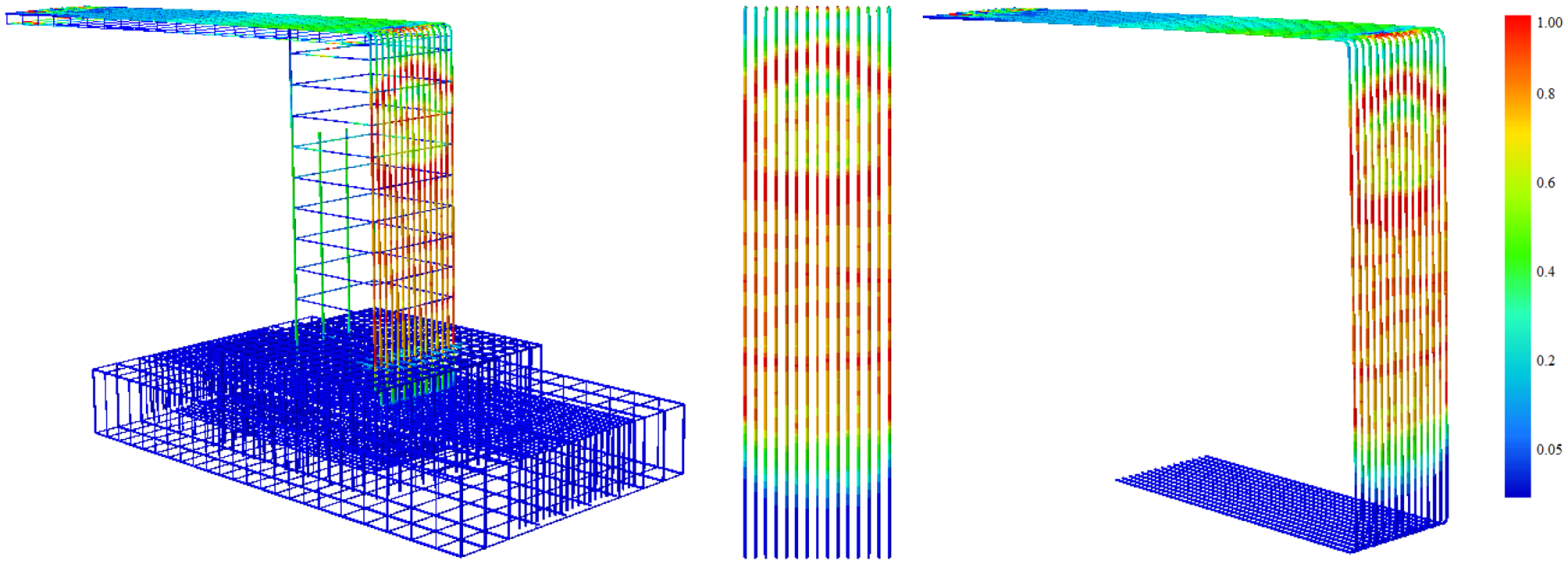
Reinforcement stress intensity.

As for the steel part, Fig. 12 shows stresses intensity in the reinforcement bars at the failure stage. The red-coloured parts of the bars were exceeded their yield strength valued by *f*_*y*_ = 435 MPa, causing the failure of the structure that was located in the tension part of the column reinforced by $\upphi$ 16 bars. As the failure happened in the location of the yielded reinforcement, a novel method is applied in this research to control the plastic deformations in these steel bars under different parameters. It is worth noting that the steel beam did not reach the yielding condition until failure, therefore it was excluded from the plastic deformations control method used in this study.

## Analysis optimization problem

This section develops the mathematical method for determining the composite bridge model's optimal loading value^17^. The maximum optimum plastic loading applied to the bridge structure ${F}^{pl}$ is determined using a nonlinear optimization approach, without the need for incremental updating of the constitutive components. ${A}_{i}$ and ${l}_{i}$ represent the cross-sectional area and length of each element respectively.

13a $$Max. \to {F}^{pl}$$

13b $${{{Subjected\,to}}: {{N}}}^{{{e}}{{l}}}= {F}^{-1}{G}{K}^{-1}{P}_{0};$$

13c $$-\overline{{N }^{pl}} \le {N}^{Pl} \le \overline{{N }^{pl}};$$

13d $$\frac{1}{2\mathrm{E}} {\sum }_{\mathrm{i}=1}^{\mathrm{n}}\frac{{l}_{i}}{{A}_{i}}{ N}_{i}^{{R}^{2}}\le {W}_{p0}.$$

**Figure 13:**
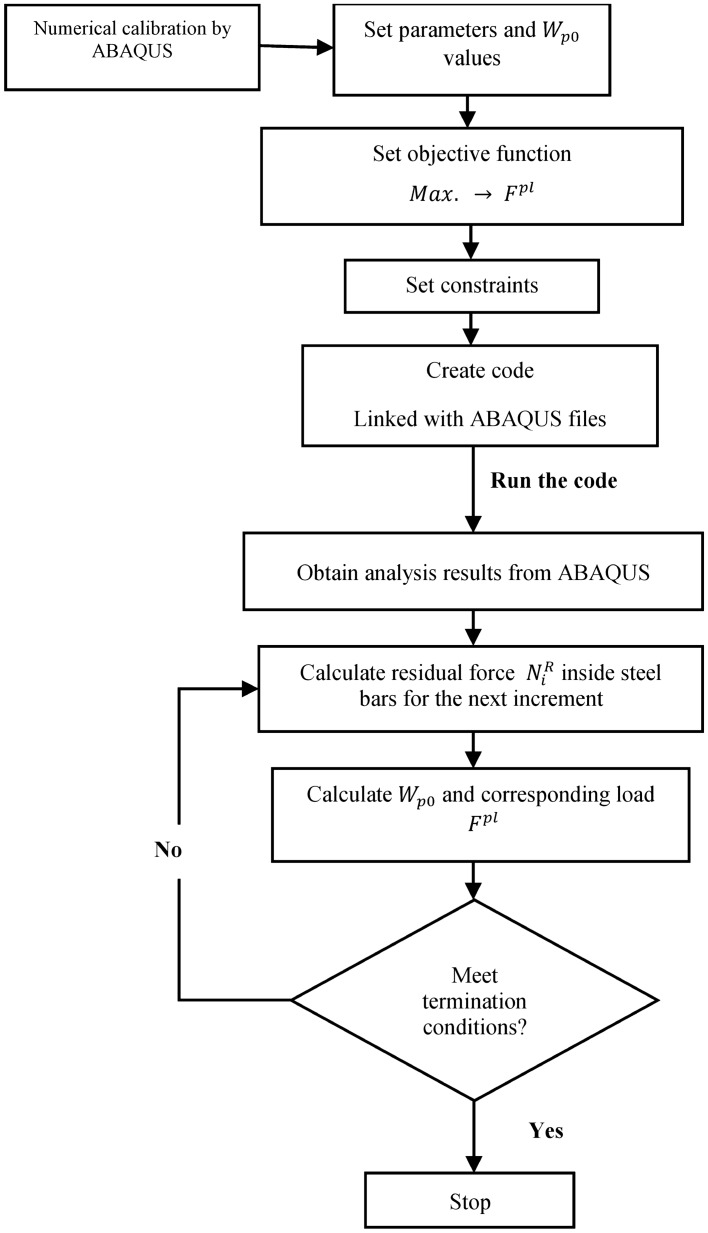
Optimization process.

Equation (13b) computes the elastic fictitious internal normal forces, while inequality Eq. (13c) indicates the lower and upper plastic limit conditions, where $\overline{{N }^{pl}}$ is the ultimate plastic limit load. Additionally, boundary Eq. (13d) illustrates the complementary strain energy of residual forces used to control the plastic deformations of steel bars as a general indicator of the structure’s plastic behaviour. The approach to solving this optimization issue is depicted in Fig. 13.

In this part, the optimum analysing solution is applied to find the maximum plastic load by applying the equations mentioned above, and to achieve this case, the residual internal stresses inside the steel bars are obtained by ABAQUS. Then, the role of the code here comes by taking these values and applying the equations to produce the complementary strain energy and comparing it with the allowed one entered in the code then the corresponding applied load is achieved. It can be said that a zero state of complementary strain energy represents the absence of any yielded steel elements, meaning that the steel is still in an elastic state and this may be accompanied by the appearance of some cracks in the concrete, but it certainly means that the structure has not yet failed or has not reached the state of plasticity, and thus the effectiveness of this method is understood, as to prevent plastic failure, a specific value of the complementary strain energy must be considered, and then examining the produced load, which will represent the maximum bearing capacity of the structure before entering the state of plasticity and failure.

**Figure 14:**
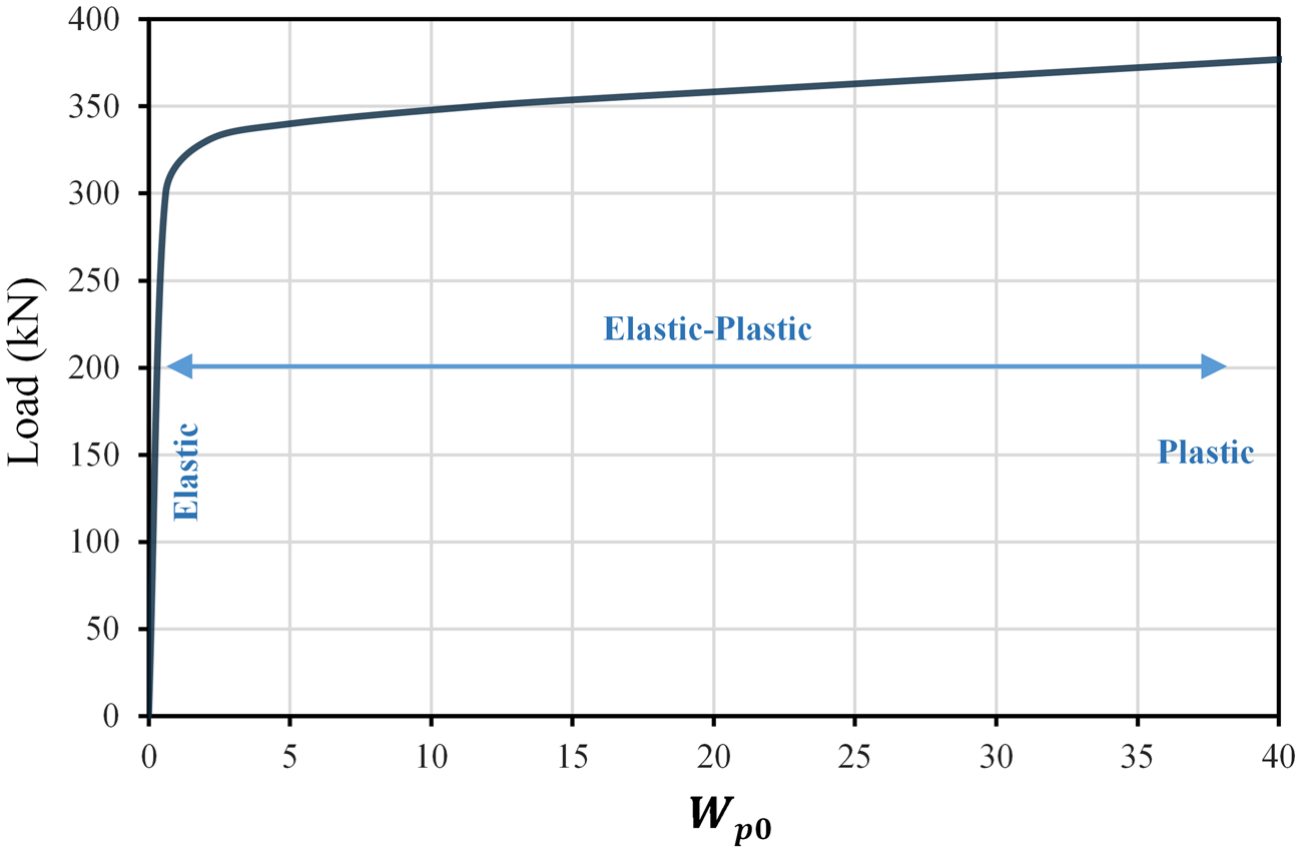
Load-complementary strain energy relation.

By applying the optimization problem mentioned above on the model tested experimentally and validated in details provided in Sections “Experimental work” and“Finite element model validation”, results are obtained and illustrated in Fig. 14, which represents the relationship between permissible complementary strain energy ${W}_{p0}$ and the load applied to the structure, and it can be understood that the structure behaves in an elastic manner until a load equal to approximately 200 kN, which is resulted by ${W}_{p0}$ value close to zero. Gradually and with an increase in the permissible complementary strain energy value ${W}_{p0},$ it is noticed that the curve begins to take a curved behavior and almost reaches the horizontal state, and this is evidence of the change in the behavior of the structure from elastic to elastic–plastic that would occurs under higher loads, where it can be declared that acquiring a state of complete horizontality is referring to the accumulation of yield stresses inside the steel bars (i.e. an increase in the value of ${W}_{p0}$) with no change in the corresponding load value (the capacity of the structure) and so this is what defines the failure.

Also, and to clarify the effect in a real and observable way, Table 3 shows the influence of the permissible complementary strain energy value ${W}_{p0}$ on the behavior of the general structure, as the first and second columns show the values of load and ${W}_{p0}$, respectively, while the third and fourth columns show the intensity of damage in both steel and concrete. It is clear that by increasing ${W}_{p0}$ value, higher loads are produced, and thus the damages caused within the steel bars and concrete are more severe and thus it reflects the plastic damage inside the structure by producing more red regions, which show regions of high stress intensity in steel and tension-damaged areas in concrete. Whereas blue colour refers to the undamaged parts (low-stress severity) and red colour refers to the entirely damaged sections (high-stress severity), that the intensity of damaged areas and stress changes by the colour.

**Table 3:** ${W}_{p0}$—load effect on the damage intensity.

${\mathrm{W}}_{\mathrm{p}0}$ $(\mathrm{N}.\mathrm{mm})$	P (kN)	Stress intensity ($\upsigma /{\upsigma }_{\mathrm{y}})$	Cracks patterns
0.5	250		
2.5	330		
10	350		
20	360		
50	388		

## The problem of the design optimization

To identify the optimal amount of reinforcing bars to use in the column section of the composite bridge model, this section develops the mathematical formula^17^. The composite bridge model's minimum steel volume is established using a nonlinear optimization approach $(V)$. The extreme concepts of plasticity can be used to avoid the need for gradual updates to the constituent parts. Additionally, ${A}_{i}$ and ${l}_{i}$ stand for each element's cross-sectional area and length, respectively.

14a $$Min. \to \mathrm{V}={\sum }_{i}{A}_{i}{l}_{i}$$

14b $$Subjected to: {N}^{el}= {F}^{-1}G{K}^{-1}{P}_{0};$$

14c $$-\overline{{N }^{pl}} \le {N}^{Pl} \le \overline{{N }^{pl}};$$

14d $$\frac{1}{2\mathrm{E}} {\sum }_{\mathrm{i}=1}^{\mathrm{n}}\frac{{\mathrm{l}}_{\mathrm{i}}}{{A}_{i}}{ N}_{i}^{{R}^{2}}\le {W}_{p0}.$$

**Figure 15:**
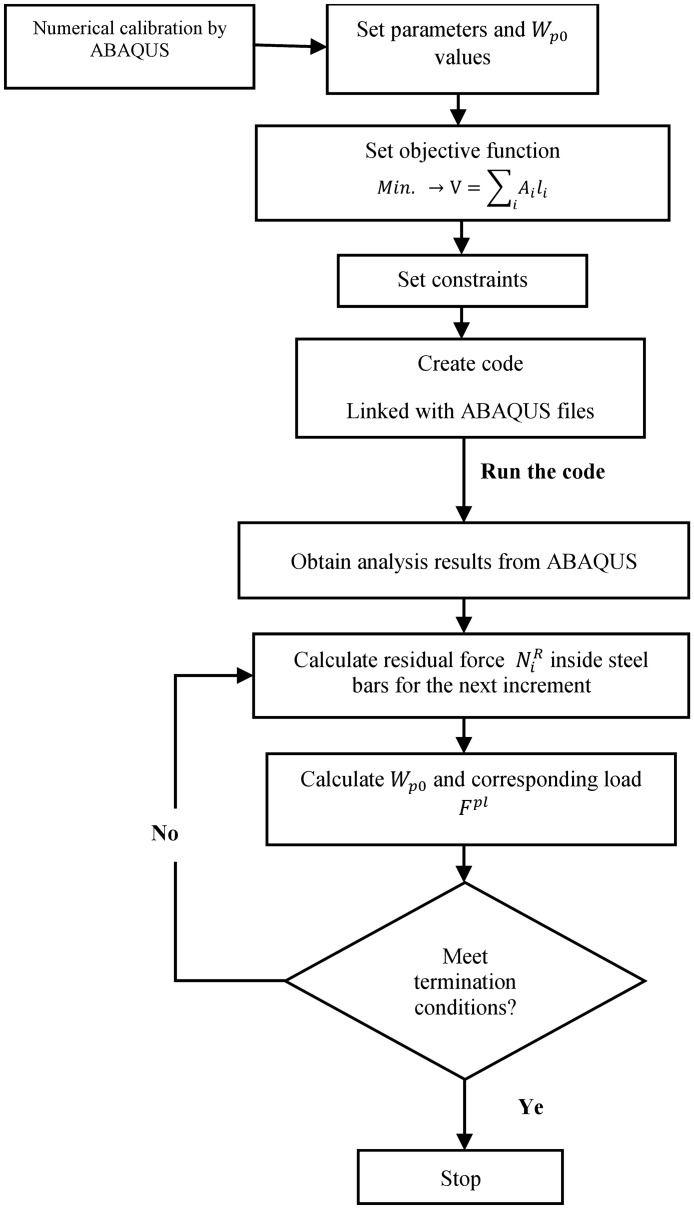
Design optimization process.

Equations (14b, c, d) have the same purposes as Eqs. (14b, c, d). In Fig. 15, the method of the design optimization problem is explained.

In this part, the optimum design case was taken into account, while the load value was kept at 300 kN, and the number of steel bars used in reinforcing the column section of the bridge is considered variable, the number of bars ranged from six bars -as a minimum- to reach 15 bars which is the original reinforcing condition^28^. As mentioned in the optimum analysis part, the internal stresses of these bars were obtained by ABAQUS and then used within the code that was provided with the above-mentioned equations to gain the complementary strain energy value and compare it with the permissible value stated inside the code then producing the load corresponding to that value and this process was done for different states of bars number and for each running increment.

**Figure 16:**
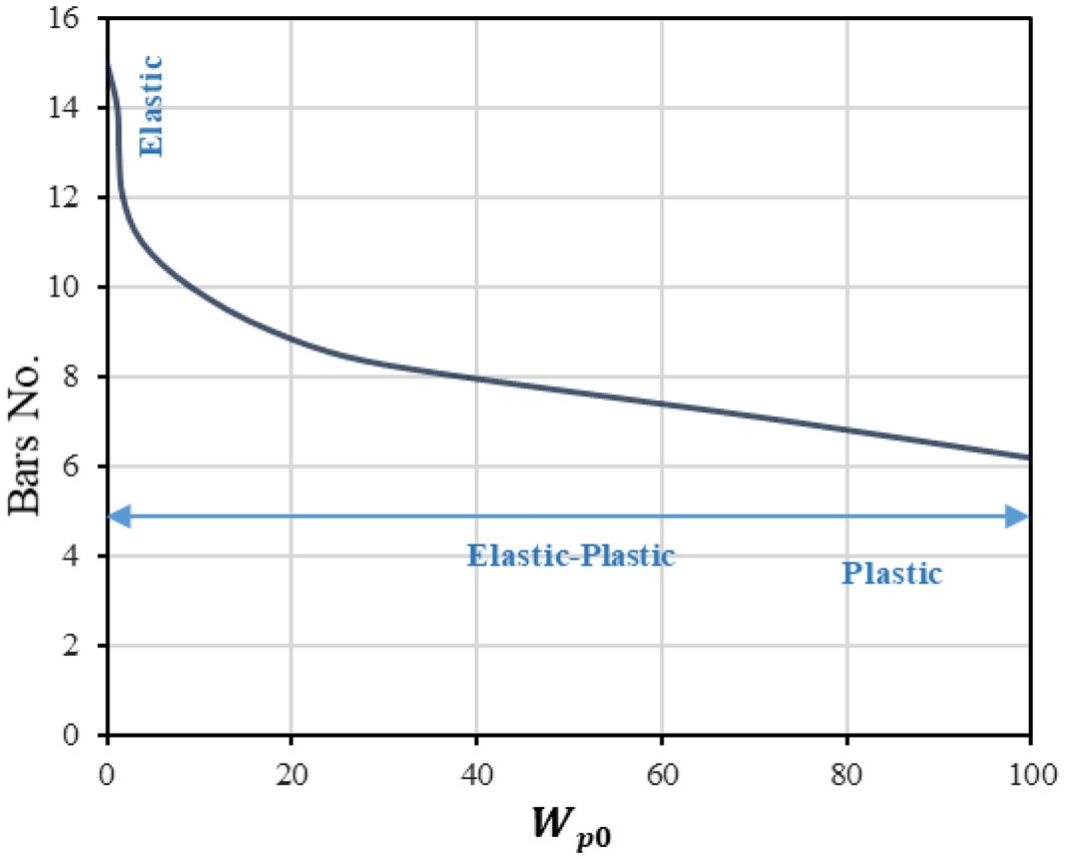
Bars No.-complementary strain energy relation.

This process was used to investigate the effect of complementary strain energy on the optimal plastic behavior of bridge structures with different bar numbers, where large quantities of reinforcing bars are required in conventional bridges to ensure sufficient strength, while excessive reinforcement bars result in excessive strength. As a result of applying the optimal design explained above, the results shown in Fig. 16 were obtained, which represent the relationship between the number of steel bars and the permissible complementary strain energy ${W}_{p0}$. The same behavior can be observed for the curve as it is almost vertical in the elastic phase and is oriented in the horizontal direction to reflect the plastic behavior of the structure. It is also noted that the zero value of ${W}_{p0}$ reflects the highest number of bars, which is evidence that providing the structure with a larger ratio of steel keeps it in the elastic state until around 300 kN, however, it can be remarked that when the value of ${W}_{p0}$ increases, this corresponds to a larger number of bars, as the larger input ${W}_{p0}$ value is met if only six bars are used, which means that when applying 300 kN, the stresses inside the six bars are higher and that the structure has entered the state of plastic deformations.

As an addition, Table 4 has been provided for a clearer explanation of the relationship of the permissible complementary strain energy ${W}_{p0}$ with the number of steel bars provided at a given load, as the first and second columns of the table represent the ${W}_{p0}$ values and the number of bars used, respectively, while the third and fourth columns represent the damage of the steel bars and concrete. In general, it can be noted that the use of fewer bars leads to the spread of red areas more severely, which represents damage, whether in steel or concrete, in contrast to the case of using bars with a larger number, and this logically represents what happens when using lower reinforcement ratios, as the structure will suffer higher stresses under lower loads if compared to higher reinforcement ratios, and this is what leads to its failure on lower corresponding loads.

**Table 4:** ${W}_{p0}$ – Reinforced bars No. effect on the damage intensity.

${W}_{p0}$ $(\mathrm{N}.\mathrm{mm})$	Bars No	Stress intensity ($\sigma /{\sigma }_{y})$	Cracks patterns
105	6		
73	7		
37	8		
18	9		
9	10		
4	11		
1.7	12		
1.4	13		
1.2	14		

I is worth mentioning that a sensitivity analysis of the optimization parameters was performed in this study to assess their influence on the performance of the elasto-plastic analysis and design of the composite integral abutment bridge. Specifically, the effects of varying complementary strain energy values on the objective function were explored. The impact of other optimization parameters, such as the maximum allowable force and the minimum number of reinforcing steel bars, was also investigated to identify the most critical parameters that significantly affect the performance of the structure. The sensitivity analysis helped in limiting the values of the inputs and to validate the optimization results and identify the most critical parameters that should be considered in future design and analysis of similar structures.

## Conclusions

This work presents a novel approach to the optimal analysis and design of a composite integral bridge under monotonic loading. The approach employs the concrete damage plasticity (CDP) constitutive model to validate the numerical model based on experimental data. In a series of optimization problems involving plastic loading and the quantity of reinforcing steel bars, the plastic deformations are constrained by the complementary strain energy of the residual internal forces. Overall, this work presents a novel approach to the optimal analysis and design of a composite integral bridge under monotonic loading. The use of complementary strain energy constraint in optimization problems provides valuable insights into the behaviour and failure of the structure. As a result, the following conclusions about this work are possible:

- The introduced method in this work effectively prevents plastic failure by maintaining a specific value of complementary strain energy, which indicates the absence of yielded steel elements and an elastic state of the structure. This is a novel approach that ensures the structure does not reach the state of plasticity, even with the presence of some cracks in the concrete.
- The study found that the bridge structure initially behaves elastically with a ${W}_{p0}$ value close to zero. However, as the ${W}_{p0}$ value increases, the structure shifts from elastic to elastic–plastic behaviour, which occurs under larger loads. The complete horizontality of the ${W}_{p0}$-load curve represents the build-up of yield stresses within the steel bars without any change in the structure's load capacity. This build-up of yield stresses ultimately determines the failure of the structure.
- As the value of ${W}_{p0}$ increases, the loads generated within the structure also increase, leading to more severe damage in both the steel bars and concrete. This increase in damage is reflected by the appearance of more red regions in the structure, indicating areas of high-stress intensity in steel and tension-damaged areas in concrete, thereby demonstrating the presence of plastic damage inside the structure.
- It is worth noting that a zero value of ${W}_{p0}$ corresponds to a higher number of reinforcing bars, indicating that a higher steel ratio can maintain the structure's elasticity until a high applied load. However, as the value of ${W}_{p0}$ increases, this corresponds to a smaller number of bars, as the larger input ${W}_{p0}$ value can only be achieved with fewer bars, which leads to higher stresses inside these bars under the same loading value and the structure entering the state of plastic deformations.
- Generally, it is observed that using fewer bars leads to more severe spreading of the red areas, indicating damage in either the steel or concrete. This is in contrast to using more bars, which can prevent or reduce the spread of these red areas. This is similar to what happens when lower reinforcement ratios are used, as the structure experiences higher stresses under lower loads compared to higher reinforcement ratios, leading to failure at lower corresponding loads.

The method presented in our paper is specifically designed for plastic analysis and the design of structures that exhibit residual stresses. The complementary strain energy approach has proven to be effective in a variety of structural types, and in our research, it is used as a means of limiting structural failure. While the scope of our paper is limited by these factors, future work could explore the application of our method to other mechanical experimental tests or reinforced concrete structures. Additionally, various optimization problems could be considered or merged to further enhance the performance of our approach.

## Data Availability

The datasets generated and analyzed during the current study are available in the main manuscript; any additional details can be obtained from the Authors.
